# Labial-gland artificial intelligence model screening for autoimmune thyroiditis among patients with connective tissue disease

**DOI:** 10.3389/fimmu.2026.1861419

**Published:** 2026-06-23

**Authors:** Jia-yun Wu, Yuening Kang, Xiao-min Li, Wen-qi Xia, Ru-yi Liao, Zhi-yang He, Yu-ling Chen, Ya Wen, Fan-xuan Meng, Jing-yu Zhang, Zheng Yang, Yong Ren, Qing Lv

**Affiliations:** 1 Department of Rheumatology, The Seventh Affiliated Hospital of Sun Yat-Sen University, Shenzhen, China; 2 Department of Pathology, The Seventh Affiliated Hospital of Sun Yat-Sen, University, Shenzhen, China; 3 Smart Healthcare Research Institute, Xunfei Healthcare Technology Co. Ltd, Hefei, China; 4 Pazhou Lab, Guangzhou, China; 5 University of Electronic Science and Technology of China, Chengdu, China

**Keywords:** autoimmune thyroiditis, connective tissue disease, deep learning, labial gland, whole-slide images

## Abstract

**Objective:**

The aim of this study is to construct a deep learning-based prediction model to accurately predict the risk of autoimmune thyroiditis (AIT) in patients with connective tissue disease (CTD) using whole section images (WSI) of labial gland pathological tissue.

**Methods:**

This was a retrospective study. The labial gland pathological sections of total 121 CTD patients were collected. According to the results of thyroid autoantibodies, including thyroglobulin antibody (TgAb) and thyroid peroxidase antibody (TPOAb), the patients were divided into positive group (Ab+ Group) and negative group (Ab- Group). The pre-trained model EfficientNet-B5 was used to extract image features, and combined with multi-instance learning and ensemble learning techniques, the high-risk prediction model for CTD patients with AIT was constructed.

**Result:**

The integrated model showed excellent prediction performance in both the internal validation set and the external validation set, with the area under the receiver operating characteristic curve (AUC) of 0.829. At the same time, the model can effectively identify the key pathological features of labial gland tissues related to the high risk of AIT in CTD patients.

**Conclusion:**

This study confirmed that the prediction model of labial gland WSI based on deep learning had good efficacy in evaluating the risk of AIT in CTD patients, which provided a new technical support and theoretical basis for early clinical identification of high-risk groups and optimization of diagnosis and treatment decisions.

## Introduction

Autoimmune thyroiditis (AIT) is an organ-specific autoimmune disease (AD) with specific thyroid autoantibodies. Multiple studies have shown that the risk of developing AIT is significantly increased in patients with connective tissue diseases (CTD), such as Sjogren’s disease (SjD), systemic lupus erythematosus (SLE), undifferentiated connective tissue disease (UCTD) and rheumatoid arthritis (RA) (1–3). As a chronic systemic ADs, CTD can affect multiple organs and systems, mainly involving exocrine glands, joint, lung, kidney, nervous system, blood system, endocrine system and other organs. As we found in our previous study, patients with SjD are prone to AIT (4). Previous study has confirmed that AIT increases the risk of thyroid Mucosa- associated lymphoid tissue lymphoma by 67-fold, and SjD increases the risk of parotid lymphoma by 44-fold (5). Importantly, concurrent AIT is not merely a complication, but further exacerbates systemic immune dysregulation in patients with CTD. It is worth noting that several studies have shown that the incidence of AIT in SLE patients with secondary SjD is significantly higher than that in patients without SjD (6, 7). These findings remind us that SjD may play a synergistic or facilitative role in the development of AIT in CTD patients.

SjD is also considered as a B cell-mediated AD (8). In the diagnosis of SjD, labial gland biopsy (LGB) is considered as the most valuable objective test (9, 10). Meta-analysis showed that the positive rate of LGB in patients with primary SjD was 63.5%-93.7%, and the specificity was 61.2%-100.0% (11). The focus score (FS) is the core index to evaluate the pathological changes of the labial gland, which is defined as the number of lymphocyte foci in every 4 mm²tissue (≥50 lymphocyte aggregates are counted as a focus) (10). FS≥1 is considered as pathological positive, which is one of the key parameters in the diagnostic criteria. However, pathological interpretation of LGB remains highly dependent on pathologists’ extensive experience and subjective judgment, with accuracy, efficiency, repeatability and quality control becoming core issues limiting the precise diagnosis and treatment of SjD.

In recent years, machine learning (ML) technology has been increasingly applied to ADs (4, 12). Wu et al. developed a novel lightweight deep learning model termed EfficientNetSwift, which was tailored to enable accurate and automated detection of oral squamous cell carcinoma from pathological images (13). Cai et al. established a weakly supervised deep learning model IBDAIM for intestinal biopsy whole-slide pathological images (WSI) analysis, which enables high-precision screening and subtype differentiation of IBD with excellent performance and effectively assists pathologists in improving diagnostic accuracy and efficiency (14). Deep learning algorithms based on convolutional neural network have significantly improved the decision-making efficiency and accuracy of clinicians (15). In our prior study, we validated the predictive value of AI for high-risk extrathyroid organ involvement (HR-OI) in SjD patients using labial gland WSI (16). However, few studies have explored endocrine disease risk stratification in CTD, particularly AIT, based on exocrine gland pathology.

Therefore, this study aims to accurately detect the pathological features of labial glands through an AI model, explore the risk of AIT in CTD patients, and further provide clinical evidence, early identification, and intervention strategies for the co-immune mechanism of internal and external gland involvement in patients with CTD.

## Methods

### Patients

CTD patients who underwent LGB in the Seventh Affiliated Hospital of Sun Yat-sen University from January 2019 to March 2024 were included in this study. SjD patients in the included population met the 2016 ACR/EULAR classification criteria (9). Patients with other CTDs, such as UCTD, systemic sclerosis, RA, and SLE, were included if they had undergone LGB. This study was approved by the Ethics Committee of the Third Affiliated Hospital of Sun Yat-sen University, and no patients received any rewards or compensation for their participation due to the retrospective nature of this study. Patient clinical data, systematic reviews, and pathological findings were obtained from the hospital information system. The ethical approval number is II2023-254-02.

### Clinical data collection

A retrospective cohort of 121 patients (59 positive, 62 negative) was enrolled from the Seventh Affiliated Hospital of Sun Yat-sen University. All participants underwent a comprehensive clinical assessment, including clinical symptoms, laboratory examinations, basic tear secretion function and pathological sections of LGB. This provided complete clinical evidence for subsequent feature extraction. TPOAb and TgAb were determined by chemiluminescent immunoassay, with their optimal diagnostic cut-off values set at 4.11 IU/ml and 5.61 IU/ml, respectively.

### Data preprocessing

Patients diagnosed with CTD should complete LGB and thyroid-specific antibody, both TPOAb and TgAb. Patients were stratified into two groups based on the results of thyroid-specific antibody assays: the positive group (Ab+ Group), defined as seropositivity for either TPOAb or TgAb (assigned an outcome value of 1), and the negative group (Ab- Group), defined as seronegativity for both TPOAb and TgAb (assigned an outcome value of 0).

Baseline characteristics were compared using IBM SPSS Statistics 27.0. For categorical variables (e.g., gender, positivity of antibodies), the chi-square test or Fisher’s exact test was applied. For continuous variables (e.g., age), the independent samples t-test was used if data were normally distributed; otherwise, the Mann–Whitney U test was performed.

Pathological slides from all patients were digitized at 400× magnification using a high-resolution slide scanner to generate WSIs. Each WSI was partitioned into non-overlapping 512×512-pixel patches through a sliding-window strategy, yielding 66,927 valid patches. All patches inherited the diagnostic label (positive/negative) from their parent WSI to ensure label consistency.

### Dataset partitioning

Stratified partitioning at the patient level was performed to prevent data leakage. The training set comprised 89 patients (45 positive, 44 negative) with 50,865 patches (27,296 positive, 23,569 negative). The independent test set included 32 patients (14 positive, 18 negative) with 16,062 patches (9,006 positive, 7,056 negative). No patient overlap existed between training and test sets.

### Deep learning model and training

The EfficientNet-B5 convolutional neural network architecture was adopted for feature extraction and binary classification. Experiments were implemented in TensorFlow 2.1. Input data consisted of normalized 512×512-pixel RGB patches. The output layer utilized a Sigmoid activation function​to generate patch-level probability scores (range: 0–1), representing the likelihood of being positive. Binary cross-entropy​served as the loss function. Model optimization employed the Adam optimizer​with an initial learning rate of 1e−4 and a batch size of 32. Data augmentation techniques included random horizontal/vertical flipping and 90° rotations to enhance generalization.

### Inference and evaluation strategy

Evaluation was conducted at two levels. At the patch-level, a threshold of 0.5 assigned binary labels (probability ≥0.5 = positive; otherwise negative). Performance metrics included accuracy, F1-score, and area under the receiver operating characteristic curve (AUC-ROC). For patient-level diagnosis, probabilities of all patches from the same WSI were averaged to derive a WSI-level probability score. A threshold of 0.5 was similarly applied for binary classification, aligning with clinical diagnostic workflows.

### Interpretability analysis, software and visualization parameters

**Figure 1:**
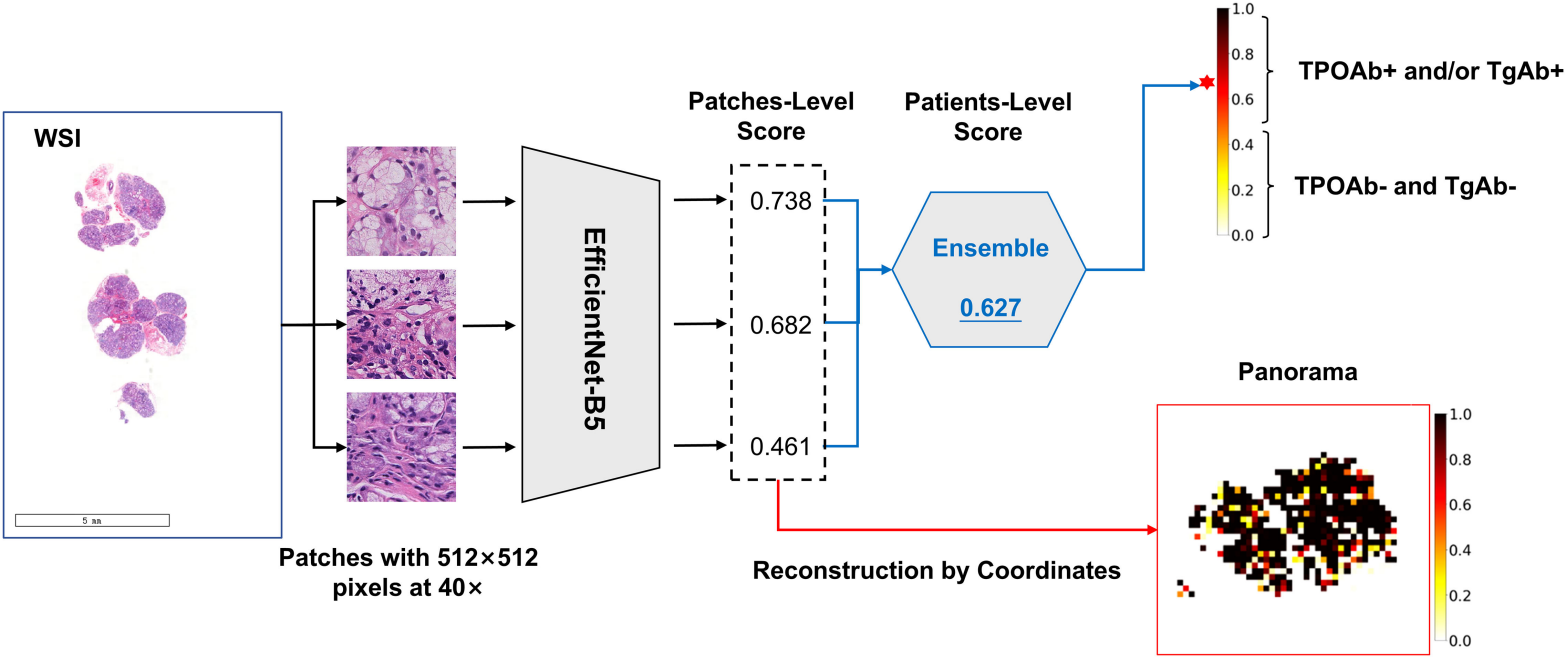
Full-process demonstration of pathological section data processing and interpretability analysis.

To visualize model interpretability, a probability-based panoramic heatmap was reconstructed for each WSI. The interpretability heatmaps were generated using custom Python scripts. Patch-level prediction probabilities were obtained from the EfficientNet-B5 model implemented in TensorFlow 2.1. Each WSI was divided into non-overlapping 512 × 512-pixel patches, and the prediction probability of each patch was recorded together with its original spatial coordinates on the WSI. The heatmap reconstruction and image overlay were performed using Python-based image processing tools, including NumPy, OpenCV, and Matplotlib. Probability values were normalized to the range of 0–1 and linearly mapped to a yellow-to-red color gradient, with light yellow representing low predicted probability and dark red representing high predicted probability. The color-coded probability map was reconstructed according to the original patch coordinates and superimposed onto the corresponding WSI thumbnail to generate the final panoramic heatmap. No manual ROI annotation or handcrafted pathomics feature extraction was used in this visualization. Color annotations were integrated and superimposed onto the WSI to generate a panoramic visualization, highlighting spatial distributions of positive/negative regions. The demonstration of pathological section data processing and interpretability analysis are shown in **Figure 1** below.

## Results

### Baseline characters of training set and independent test set

Training set (89 participants): 44 in Ab- group, 45 in Ab+ group; diagnoses included SjD (73.0%), RA (15.8%) and UCTD (11.2%). There were no significant differences in age (both 45 ± years, p>0.05), gender (females 82% vs 93%, p>0.05), clinical symptoms, organ involvement, routine laboratory parameters or autoantibody positive rates between the two groups (all p>0.05). Significant differences existed in tear function: Ab- group had lower positive rates of tear secretion test (30% vs 53%, p<0.05) and Chisholm grade ≥3 (52% vs 76%, p<0.05) (shown in **Table 1**).

**Table 1:** Characteristics, clinical and laboratory data of 89 participants in the training set with or without thyroid-specific autoantibodies.

Variable	Ab- Group (n=44)	Ab+ Group (n=45)
Age (years)	45 ± 13	45 ± 12
Female (n, %)	36 (82%)	42 (93%)
Symptoms (n, %)		
Dryness of mouth	27 (61%)	23 (51%)
Dryness of eye	28 (64%)	28 (62%)
Arthralgia	20 (46%)	26 (58%)
Skin Rash	2 (5%)	7 (16%)
Involvement of Any Organ (n, %)	28 (64%)	31 (69%)
ILD	3 (7%)	5 (11%)
Hematological System	26 (59%)	28 (62%)
Renal System	3 (7%)	3 (7%)
Nervous System	1 (2%)	2 (4%)
Cardiovascular System	0 (0%)	0 (0%)
WBC	5.11 (3.57, 6.23)	5.07 (4.62, 6.90)
RBC	4.30 (3.96, 4.59)	4.22 (3.99, 4.52)
PLT	210 (182, 243)	215 (176, 234)
AST	14 (9, 22)	17 (13, 30)
ALT	18 (15, 24)	21 (15, 27)
Cr	58.5 (52.3, 67.5)	58.0 (52.6, 65.7)
CRP	1.20 (0.47, 2.29)	1.48 (0.72, 5.73)
ESR	22 (12, 38)	24 (15, 45)
IgA (g/L)	2.58 (1.64, 3.13)	2.63 (2.08, 3.43)
IgG (g/L)	14.2 (11.8, 15.5)	15.1 (12.6, 18.8)
IgM (g/L)	1.216 (0.844, 1.780)	1.100 (0.740, 1.700)
C3 (g/L)	1.029 ± 0.218	1.039 ± 0.180
C4 (g/L)	0.216 (0.170, 0.312)	0.199 (0.167, 0.274)
RF positive (n, %)	12 (27%)	19 (42%)
Anti-CCP positive (n, %)	4 (9.1%)	9 (20%)
ANA≥1/320 (n, %)	29 (66%)	30 (68%)
SSA positive (n, %)	25 (57%)	31 (69%)
SSB positive (n, %)	15 (34%)	15 (33%)
Ro-52 positive (n, %)	23 (52%)	24 (53%)
Tear Secretion Test Positive (n, %)*	13 (30%)	24 (53%)
Chisholm grade≥3 (n, %)*	23 (52%)	34 (76%)

Remarks: *p value<0.05, ILD Interstitial Lung Disease.

Independent test set (32 participants): 18 in Ab- group, 14 in Ab+ group; diagnoses included SjD (75.0%), RA (12.5%) and UCTD (12.5%). No significant differences were found in age (49 ± 12 vs 47 ± 11 years, p>0.05) or gender (females 67% vs 93%, p>0.05). Significant differences (p<0.05): Ab- group had lower proportions of any organ involvement (28% vs 71%), hematological system involvement (28% vs 71%) and Chisholm grade ≥3 (50% vs 93%), lower IgA (1.96 ± 0.57 vs 2.61 ± 0.67 g/L) and IgG (12.7 ± 2.2 vs 16.3 ± 5.9 g/L), but higher Ro-52 positive rate (61% vs 7%). Other indicators showed no significant differences (shown in **Table 2**).

**Table 2:** Characteristics, clinical and laboratory data of 32 participants in the independent test set with or without thyroid-specific autoantibodies.

Variable	Ab- Group (n=18)	Ab+ Group (n=14)
Age (years)	49 ± 12	47 ± 11
Female (n, %)	12 (67%)	13 (93%)
Symptoms(n, %)		
Dryness of mouth	10 (56%)	7 (50%)
Dryness of eye	9 (50%)	10 (71%)
Arthralgia	14 (78%)	10 (71%)
Skin Rash	0 (0%)	0 (0%)
Involvement of Any Organ (n, %)*	5 (28%)	10 (71%)
ILD	0 (0%)	0 (0%)
Hematological System*	5 (28%)	10 (71%)
Renal System	1 (6%)	0 (0%)
Nervous System	0 (0%)	0 (0%)
Cardiovascular System	0 (0%)	0 (0%)
WBC	5.34 (3.96, 7.24)	4.43 (3.69, 6.23)
RBC	4.53 ± 0.67	4.19 ± 0.62
PLT	224 ± 55	221 ± 82
AST	16 (10, 31)	15 (10, 23)
ALT	18 (14, 21)	18 (14, 23)
Cr	66.5 ± 23.0	56.3 ± 9.8
CRP	0.88 (0.32, 4.44)	1.15 (0.50, 4.02)
ESR	12 (5, 28)	24 (12, 36)
IgA (g/L)*	1.96 ± 0.57	2.61 ± 0.67
IgG (g/L)*	12.7 ± 2.2	16.3 ± 5.9
IgM (g/L)	0.987 (0.672, 1.643)	1.160 (0.895, 1.673)
C3 (g/L)	0.958 ± 0.192	0.998 ± 0.227
C4 (g/L)	0.206 ± 0.069	0.217 ± 0.113
RF positive (n, %)	6 (33%)	3 (21%)
Anti-CCP positive (n, %)	4 (22%)	2 (14%)
ANA≥1/320 (n, %)	3 (17%)	7 (50%)
SSA positive (n, %)	13 (72%)	5 (36%)
SSB positive (n, %)	5 (28%)	2 (14%)
Ro-52 positive (n, %)*	11 (61%)	1 (7%)
Tear Secretion Test Positive (n, %)	12 (67%)	7 (50%)
Chisholm grade≥3 (n, %)*	9 (50%)	13 (93%)

Remarks: *p value<0.05, ILD Interstitial Lung Disease.

### Classification performance at the patch level

**Figure 2:**
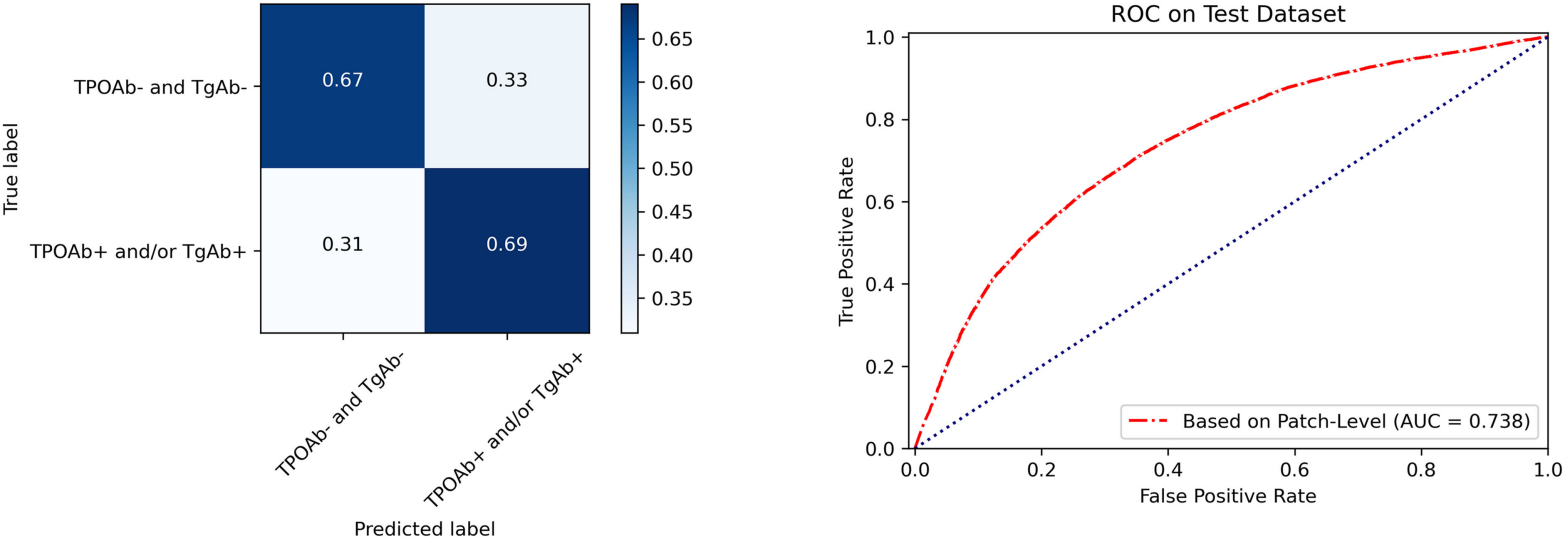
ROC curve for patch-level classification performance in the independent test set.

The deep learning model demonstrated moderate discriminative capability​on the test set comprising 16,062 patches, including 9,006 in Ab+ Group and 7,056 in Ab- Group. Key performance metrics were as follows: area under the curve (AUC) = 0.738, F1-score = 0.68, and accuracy (ACC) = 0.68. Further analysis revealed that the model exhibited stronger performance in identifying positive patches, with a positive predictive value (PPV) of 0.72 and sensitivity (SENS) of 0.69. The AUC values and other performance metrics of patch-level are shown in **Figure 2** and **Table 3** below.

**Table 3:** The AUC values and other performance metrics for deep learning model in test set.

Analysis level	AUC	F1	ACC	PPV	NPV	SENS	SPEC
Patch Level	0.738	0.68	0.68	0.72	0.63	0.69	0.67
Patient Level	0.829	0.75	0.75	0.71	0.78	0.71	0.78

ACC accuracy, PPV positive predictive value, NPV negative predictive value, SENS sensitivity, SEPC specificity.

### Diagnostic performance at the patient level

**Figure 3:**
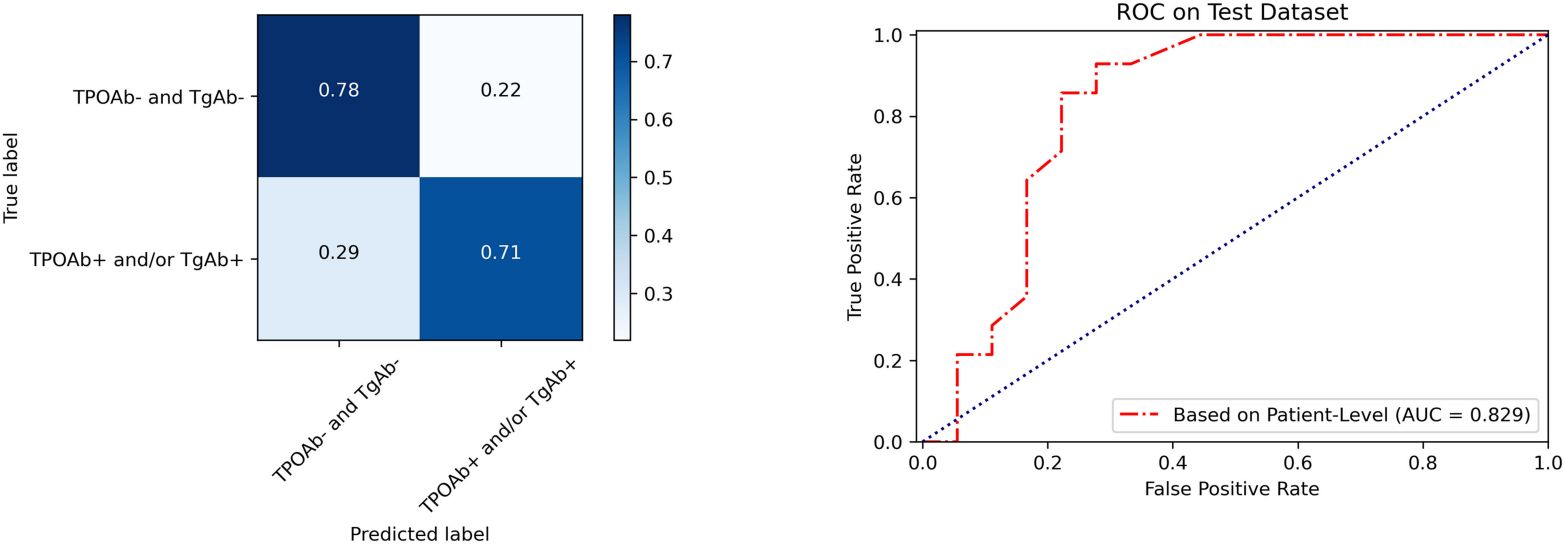
ROC curve for patient-level diagnostic performance in the independent test set.

Aggregation of patch-level predictions (by averaging probabilities per WSI) yielded significantly enhanced diagnostic performance​at the patient level. On the independent test set of 32 patients (14 positive, 18 negative), the model achieved an AUC of 0.829, F1-score of 0.75, and ACC of 0.75. Notably, the model demonstrated high reliability in excluding negative cases, with an NPV of 0.78 and SPEC of 0.78. Nevertheless, improvement opportunities remained for positive case identification, as sensitivity (SENS) was 0.71 and PPV was 0.71. This demonstrates that patient-level diagnosis improves overall performance, particularly in ruling out negative cases, while further optimization is warranted for accurately detecting positive cases. The AUC values and other performance metrics of patient-level are shown in **Figure 3** and **Table 3** below.

### Explanation of model interpretability

**Figure 4:**
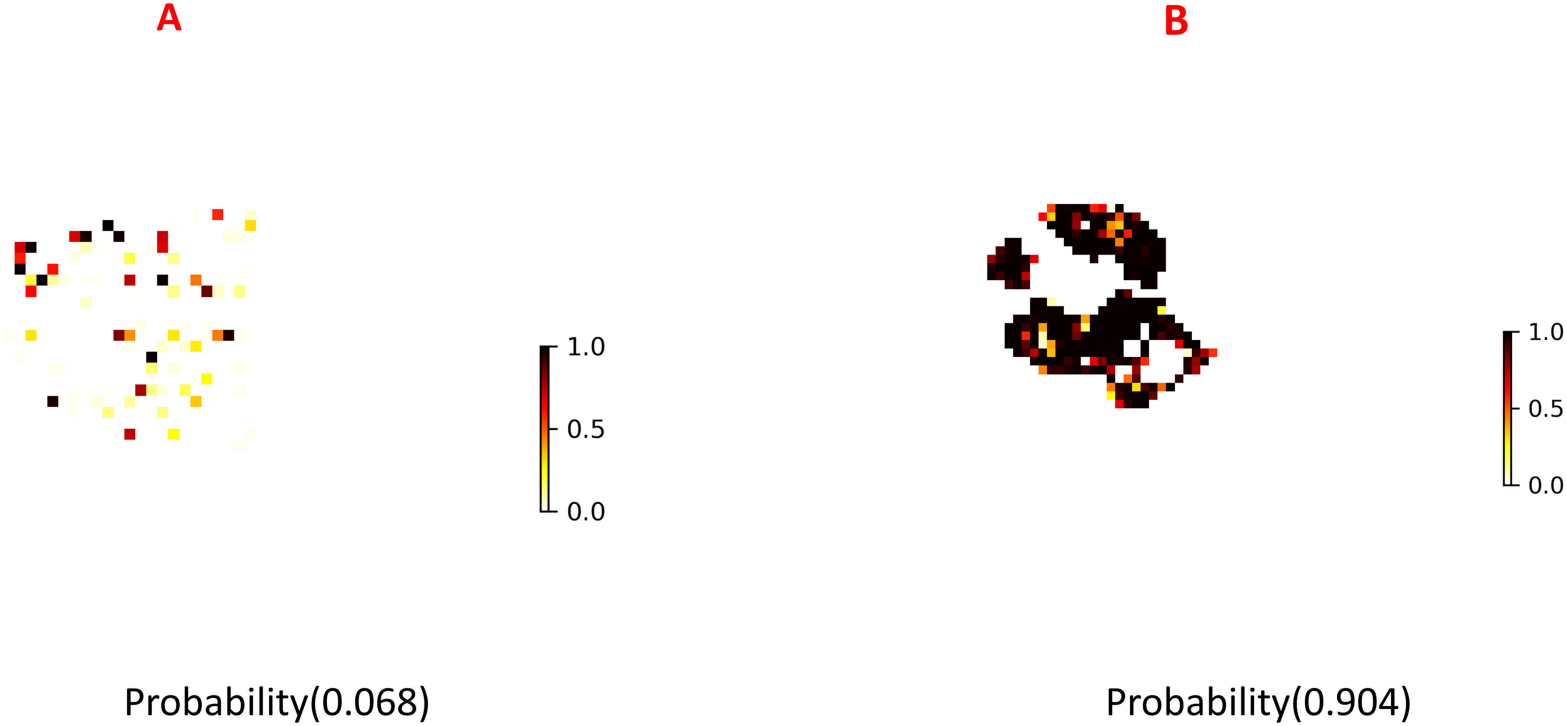
This figure shows the heatmap generated by the model for WSI of labial gland pathology from two enrolled patients. Heatmaps of Patient **(A)** (low AIT predictive probability, 0.068) and Patient **(B)** (high AIT predictive probability, 0.964) from the independent test set.

The model generated a probability for each tissue patch and integrated it with the patch’s spatial coordinates on the original WSI to construct a predictive heatmap. As shown in **Figure 4**, Patient A in the independent test set (overall predictive probability=0.068) presented a heatmap with diffuse pale yellow low-intensity signals and no clustered dark red areas, indicating the model identified few AIT-relevant tissue regions and a low AIT risk. Conversely, Patient B (overall predictive probability=0.964) showed distinct clustered dark red high-probability regions in the heatmap, reflecting the model’s effective localization of AIT-associated pathological areas and a high AIT risk. It should be noted that the heatmap does not represent manually annotated regions of interest or predefined handcrafted pathomics features. Instead, it visualizes the spatial distribution of model-derived patch-level prediction probabilities, thereby indicating regions that contributed more strongly to the WSI-level prediction. Clinically, Patient A had TgAb and TPOAb levels both <3.00 IU/mL, while Patient B had TgAb 115.29 IU/mL, TPOAb 711.68 IU/mL, reduced TSH and elevated FT3/FT4 (consistent with hyperthyroidism). This consistency verified that the EfficientNet-B5 model could reliably identify and localize AIT-related pathological features, providing an interpretable visual foundation for auxiliary AIT diagnosis.

## Discussion

In our study, the mean age of onset of CTD with TPOAb or TgAb was 45 to 50 years, which is consistent with the mean age of onset of Hashimoto’s thyroiditis(HT) reported previously (17). The majority of CTD patients with or without AIT were female, but the proportion of female patients in the Ab+ group was higher, but the difference was not statistically significant. This result is also consistent with previous reports that the female-to-male ratio is at least 7:1 to 10:1 in HT patients (18).

Our study showed that the deep learning model based on labial salivary gland biopsy pathological slices achieved excellent predictive performance for AIT in CTD patients, even without integration of clinical baseline data. This is the first report on predicting AIT via a deep learning model using key features from WSI images of labial gland biopsies in CTD patients. The AI model was validated to automatically identify the key features in WSI images and establish the association between these features and the risk of AIT. In addition, the model(AUC = 0.829) based only on the pathological characteristics of labial gland biopsy was more effective than that based on clinical data in our previous study, with the random forest model achieving an AUC of 0.755 (4). Machine learning’s clinical utility is advancing rapidly. For example, prior studies built models with ResNet-50 and EfficientNet as backbones to analyze X-ray images, achieving excellent classification of osteoporotic vertebral compression fractures (19, 20). Another study trained an EfficientNet-b5 model on 28,155 double-balloon enteroscopy images from 628 patients for lesion detection and multidimensional ulcer grading in small-bowel Crohn’s disease (21). These studies have shown that AI models constructed based on ML exhibit higher accuracy, practicality and generalizability in disease diagnosis and prediction.

Studies have found that the positive rate of thyroid antibodies in the general population is about 7.1% (22). Prior studies have indicated that the risk of concurrent AIT in SjD patients reaches 30%-40% (23, 24), and this increased risk is also observed in patients with other types of CTD (25, 26). From these studies, it can be speculated that CTD in some way significantly increases the risk of AIT. Notably, thyroid function is normal in the majority of patients who are positive for TPOAb. Hypothyroidism occurs in only 20% to 30% of patients with HT (27). In a trial involving 248 initially euthyroid patients with positive TPOAb, only 12.5% developed subclinical and overt hypothyroidism or hyperthyroidism after 3 years of follow-up (28). The diagnosis of AIT comorbidity primarily relies on screening through serological specific antibodies (TPOAb, TgAb), thyroid function tests, and thyroid ultrasound. Under such circumstances, large-scale screening for AIT undoubtedly increases the economic burden on patients. The model in our study can utilize existing objective pathological sections from patients’ labial salivary gland biopsies to directly stratify risk in this patient population on the basis of labial gland pathology, effectively avoiding waste of medical resources and economic burden associated with large-scale screening, thereby demonstrating significant clinical utility.

This study identifies a positive correlation between specific WSI features of CTD patients and AIT risk, implying a shared immune mechanism underlying endocrine and exocrine gland involvement in CTD. Our findings further support the conclusion that different ADs are universally associated and may share a common pathogenesis involving impaired immune self-tolerance (29, 30). For instance, both SLE and AIT are characterized by predominant Th1 immunity and share common key cytokines and chemokines including IFN−γ, TNF−α, and CXCL10, which constitute the fundamental immunopathological basis for their close association (31). Similarly, SjD and AIT share highly similar pathogenesis, including CD4+ T lymphocyte infiltration, abnormal B−cell activation, shared HLA susceptibility genes, and glandular injury mediated by CXCL10 (32). AIT is pathologically characterized by massive lymphoplasmacytic infiltration in thyroid tissue, mainly CD4^+^T cells, CD8^+^T cells, and B cells, which can form lymphoid follicle-like structures (33–35). Similarly, LGB in patients with CTD shows characteristic lymphocytic infiltration, including CD4 + Th1 cells, CD8 + cytotoxic T cells, and autoantigen-producing B cell in salivary gland lesions of patients with SjD patients (36), highly homologous to thyroid tissue of AIT. The consistency in lymphocyte subsets, functional phenotypes and aggregation patterns between the lesional tissues of these two ADs directly reflects their shared core mechanism of immune dysregulation. Lymphoplasmacytic infiltration degree, follicular structure formation and other LGB features can serve as pathological markers for AIT prediction, and exocrine gland local immune microenvironment status may offer clues for exploring the two ADs immune mechanisms. Notably, 73% of our cohort were SjD patients, suggesting SjD-specific labial gland pathological features (lymphocyte infiltration, acinar destruction, fibrosis, etc.) may correlate more closely with high AIT risk. Larger multicenter studies across different CTD subgroups are required to clarify the specific associations between distinct CTD and AIT.

This study has several limitations. First, the small sample size may reduce the model’s external validity; future multicenter clinical studies are needed to expand the sample size and improve its generalizability. Second, no subgroup analysis was conducted for different CTD subtypes, precluding further exploration of differences in the shared immune mechanisms between various CTD and AIT, which will be a key focus of subsequent research. Although the probability-based heatmap provided spatial information regarding model-derived high-risk regions, it did not directly correspond to specific predefined histopathological structures or handcrafted morphological features, such as nuclear morphology, cytoplasmic density, cellular outline, glandular epithelial changes, or tissue microenvironmental characteristics. This remains an inherent limitation of the current weakly supervised deep learning framework. In future studies, more fine-grained pathomics approaches could be integrated with WSI-based deep learning, including nuclear and cellular segmentation, quantitative morphological feature extraction, texture analysis, glandular structure analysis, lymphocyte infiltration assessment, and spatial distribution analysis. These approaches may help identify interpretable pathological features, such as nuclear size and shape, cellular density, inflammatory cell aggregation, acinar destruction, ductal epithelial changes, fibrosis, and lymphoid follicle-like structures. Such interpretable feature mining may further improve the biological explainability of the model and provide more clinically actionable pathological indicators for AIT risk stratification in patients with CTD.

## Conclusions

In this study, we established a predictive model based on EfficientNet-b5 using WSI of LGB specimens from patients with suspected SjD among CTD. The results demonstrated that this model is capable of automatically identifying key pathological features in WSI images and exhibits a high performance in assessing the risk of AIT comorbidity in patients with CTD, especially SjD. It is well-documented that CTD and AIT exhibit shared immune mechanisms.

## Data Availability

The original contributions presented in the study are included in the article/supplementary material. Further inquiries can be directed to the corresponding authors.
